# Developing a Colorimetric Equation and a Colorimetric Model to Create a Smartphone Application That Identifies the Ripening Stage of Lady Finger Bananas in Thailand

**DOI:** 10.3390/s23146387

**Published:** 2023-07-13

**Authors:** Bhoomin Tanut, Watcharapun Tatomwong, Suwichaya Buachard

**Affiliations:** 1 Department of Computer Science and Information Technology, Faculty of Science and Technology, Kamphaeng Phet Rajabhat University, Kamphaeng Phet City 65000, Thailand; bhoomin_t@kpru.ac.th; 2 Wireless Engineering Department, National Telecom Public Company Limited, Bangkok 10210, Thailand; watcharapun.t@ntplc.co.th; 3 Department of Biology, Faculty of Science and Technology, Kamphaeng Phet Rajabhat University, Kamphaeng Phet City 65000, Thailand

**Keywords:** colorimetric equation, colorimetric detection model, COCO-SSD object detection model, automatic power-law transformation method

## Abstract

This article develops a colorimetric equation and a colorimetric model to create a smartphone application that identifies the ripening stage of the lady finger banana (LFB) (*Musa AA group ‘Kluai Khai’*, กล้วยไข่ “gluay kai” in Thai). The mobile application photographs an LFB, automatically analyzes the color of the banana, and tells the user the number of days until the banana ripens and the number of days the banana will remain edible. The application is called the Automatic Banana Ripeness Indicator (ABRI, pronounced like “Aubrey”), and the rapid analysis that it provides is useful to anyone involved in the storage and distribution of bananas. The colorimetric equation interprets the skin color with the CIE L*a*b* color model in conjunction with the Pythagorean theorem. The colorimetric model has three parts. First, COCO-SSD object detection locates and identifies the banana in the image. Second, the Automatic Power-Law Transformation, developed here, adjusts the illumination to a standard derived from the average of a set of laboratory images. After removing the image background and converting the image to L*a*b*, the data are sent to the colorimetric equation to calculate the ripening stage. Results show that ABRI correctly detects a banana with 91.45% accuracy and the Automatic Power-Law Transformation correctly adjusts the image illumination with 95.72% accuracy. The colorimetric equation correctly identifies the ripening stage of all incoming images. ABRI is thus an accurate and robust tool that quickly, conveniently, and reliably provides the user with any LFB’s ripening stage and the remaining days for consumption.

## 1. Introduction

Bananas are a popular fruit around the world, and they belong to the genus *Musa* in the family Musaceae and are of the order Scitamineae. Bananas grow in tropical and subtropical areas of more than 130 countries. The majority of edible cultivars are allopolyploid triploids with a genome type of AAA (dessert bananas), AAB (plantains), or ABB (other cooking bananas) [1]. In 2020, 119,833,677 tonnes of bananas were produced worldwide, of which 64,725,093 tonnes or 54.4% were produced in Asia. In Southeast Asia specifically, 32,859,278 tonnes of bananas were produced, with Thailand producing 1,360,670 tonnes [2]. Bananas are one of the most widely cultivated tropical fruits, and they can be grown on small-scale farms with low production costs, yielding the first harvests 14 months after planting and continuing for up to 10 years. There are five important commercial banana cultivars in Thailand: Kluai Hom Thong, Kluai Namwa, Kluai Khai, Kluai Hakmuk, and Kluai Lep Mu Nang. Harvesting bananas provides farmers with income throughout the year [3], and bananas can be grown as a main crop or in mixed crops.

The lady finger banana (LFB) (*Musa genome subgroup AA ‘Kluai Khai’*, กล้วยไข่ “gluay kai” in Thai) has relatively small fruits that are particularly sweet and flavorful. Chanthaburi Province grows the most LFBs of any Thai province (approximately 10,173.23 acres in 2014), followed by Nakhon Sawan, Phetchaburi, Tak, Chumphon, Kamphaeng Phet, Rayong, and Sukhothai. Some LFBs are exported unprocessed to China, Japan, and Vietnam [4], while others are sent to food processing plants to be peeled, sliced, and canned in syrup. The main international markets for canned LFBs are the United States and France. Kamphaeng Phet Province has a specific type of LFB called Kluai Khai Kamphaeng Phet (KP-LFB), which are generally even smaller and sweeter than other LFBs, with a distinctive flavor. Kluai Khai Kamphaeng Phet was registered as an official geographical indication (GI) in 2016 [5]. KP-LFB have not been specially bred for convenient marketing characteristics. They tend to be more fragile than other banana types, bruising easily. As they ripen, they also produce more freckles than other banana types, which decreases their visual appeal and sale value. Thus, careful and timely transportation of KP-LFB bound for far destinations is particularly important [6]. Since freckling is a function of the ripening process, understanding ripeness levels and being able to identify them can help farmers estimate how many days remain before bananas are ripe. This, in turn, can help farmers plan ahead and sell more bananas in peak condition. It should be noted that KP-LFB handled under normal local conditions in Kamphaeng Phet Province are not temperature-controlled or humidity-controlled and are not treated with ethylene as a ripening tool. This study is not about cavendish banana varieties or the conditions under which cavendish bananas are handled in other places. This study develops a solution for a local challenge. That said, the techniques used here could be included in solutions for other challenges in other places.

Various technologies and tools for have been developed for identification of fruit ripening stages. Researchers have used spectrometers, sensors, and computer software for this purpose. These tools are well suited for laboratory investigation, because in a lab it is easier to control light interference and environmental factors that affect changes in color value. The following are examples of related research confined to a laboratory, i.e., not mobile applications. Prommee et al. [7] developed a device and software to identify and predict banana quality. They converted RGB color values to L*a*b* color values, which were then used to identify banana ripeness stages. Ringer et al. [8] used real-time in situ techniques to determine the ripening stage of bananas non-invasively. Their work involved gloss measurement on the banana skin surface using a luster sensor. This method was able to identify seven ripeness levels of bananas, from unripe (called level R1) to overripe (called level R7). Prabha, S. et al. [9] used image processing to assess banana fruit maturity, and their method can identify three stages: under-mature, mature, and over-mature. Zhu, L. et al. [10] developed a banana grading system which uses the support vector machine and YOLO algorithms. They extract three features from the image: the banana shape, the skin color (RGB converted to HSV), shape, and the skin texture. The software can identify three classes: unripe, ripe, and over-ripe. Yusuf A. R. et al.’s study [11] developed a method for the identification of cavendish banana maturity which applied convolutional neural networks (CNN) and can identify four ripening stages: unripe, almost ripe, ripe, and overripe. A. Anushya’s study [12] used Gray Level Co-Occurrence Matrix (GLCM) and classification algorithms (decision trees and neural networks) to define banana ripening stages using classifiers. The three classes are almost ripe, ripe, and overripe. A study by Zhang, Y. et al. study [13] used deep learning for the classification of bananas’ seven ripening stages. They applied convolutional neural network architecture, resulting in indicators that can help differentiate the subtle differences among subordinate classes of bananas in the ripening state (R1–R7). In contrast to research described above, the examples below were developed for use on a smartphone for convenience and portability. Intaravanne, Y. et al. [14] developed a mobile application for estimation of banana ripeness estimation that used two-dimensional spectral analysis. This method can identify three stages: immature, ripe, and overripe stage. Kipli, K. et al. [15] used Google Cloud Vision and applied an RGB color system for banana ripeness evaluation via mobile application. That application can classify the same three stages of ripeness as in the study by Intaravanne et al. [14] The previous studies mentioned above use methods that differ from each other in various ways, but one thing that most of the studies have in common is that they identify the stages of ripening in three to five stages. To date, there is no known mobile application for quickly and conveniently assessing the ripeness of lady finger bananas using image processing.

The current study develops a colorimetric mobile application that works in all light conditions because it is based on a new colorimetric equation and a new automatic illumination adjustment method called the Automatic Power-Law Transformation (APLT) method. There are three main components in the development of this application. First, a colorimetric equation is created to ret the color of the banana skin into the CIE L*a*b* color system [16]. The second component is an object detection model that uses COCO-SSD in the mobile application [17] to detect a banana within an image. The final component in the development of the application in this study is an Automatic Power-Law Transformation method applied in image processing that calculates energy and adjusts light quality. The objective is for the application to function well in any light environment with accuracy comparable to laboratory conditions. The application output consists of indication of the ripeness stage of the banana and an estimate of the remaining number of days in which it can be consumed, referred to in this study as the remaining days for consumption (RDFC), which includes the current day but not the day that the banana becomes overly ripe. The application developed here can benefit all people who work in the banana business and, by extension, the public, who can have access to quality bananas with greater reliability.

## 2. Materials and Methods

**Figure 1:**
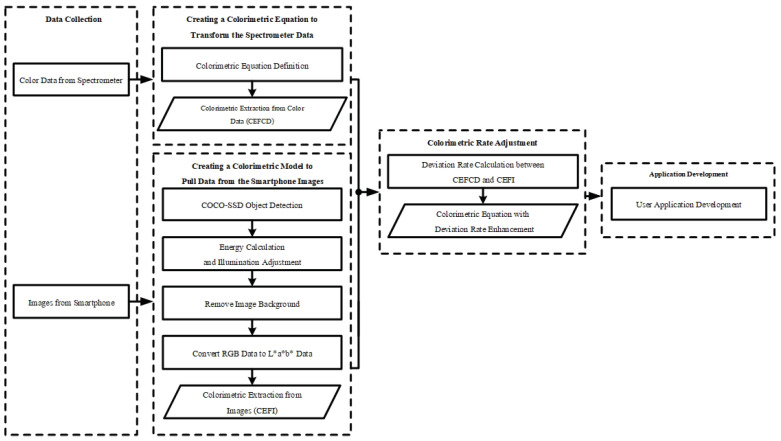
Schematic diagram of the five stages of application development.

The colorimetric equation and colorimetric application in this study have been developed using three kinds of software. A BLUE-Wave Miniature Spectrometer with SpectraWiz Spectrometer Software v5.1 [18] is used for analysis of banana skin colors to establish the colorimetric equation. MATLAB 2015B [19] is used to analyze the banana skin color in each incoming image. Finally, Visual Code Studio 2020 [20] is used to build the mobile application. Testing was carried out using a spectrometer (BLUE-Wave model, StellarNet Inc., Tampa, FL, USA) for collection of banana skin color and a smartphone (iPhone XS Max, Apple, Cupertino, CA, USA, with IOS 14.6) [21]. Figure 1 shows a schematic diagram of the five steps involved in development of this application. The five main stages are the following: data collection, colorimetric equation creation using color data, colorimetric model creation using images, colorimetric model adjustment, and application development.

### 2.1. Data Collection

**Figure 2:**
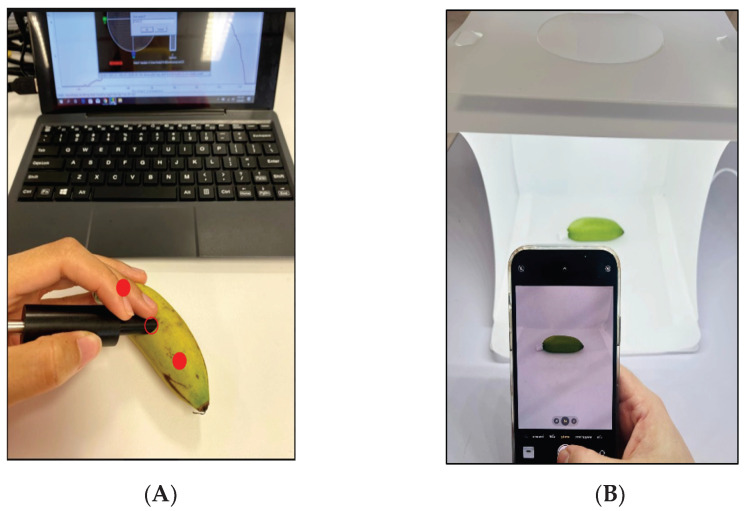
Banana skin color was captured in two ways: using a spectrometer (**A**) and using a smartphone camera with a light box (**B**).

In order to observe the ripening process of KP-LFB and divide the process into ripening stages, KP-LFB were harvested at a farm in Kamphaeng Phet City, approximately 45–50 days after the first appearance of the inflorescence (flower spike), as described by Tongpoolsomjit et al. [22]. At the time of cutting, the bananas were light green with some yellow, but still 75–80%, because as Tongpoolsomjit et al. explain, bananas cut earlier will not ripen, only shrivel. The dataset for the colorimetric equation and colorimetric application in this study comes from 80 of these harvested bananas. The changing skin color of these bananas was captured every day in two ways: first, with a spectrometer; and then, with a smartphone camera in a light box, for approximately 6–18 days until they reached stage R7, as described by Bains et al. [23]. Pictures of dathe ta collection are shown in Figure 2.

When using the spectrometer, the color was recorded at 3 points spread across the length of the banana, but the tips were avoided, as shown in Figure 2A. The results from these three points were later averaged. The smartphone pictures were taken from the open front of a light box with dimensions of 24 × 24 × 24 cm ${}^{3}$. They were equipped with a 20-watt strip of 20 tiny LED lights, as shown in Figure 2B. The average color of the smartphone image was then obtained using image processing techniques that first removed the image background.

**Figure 3:**
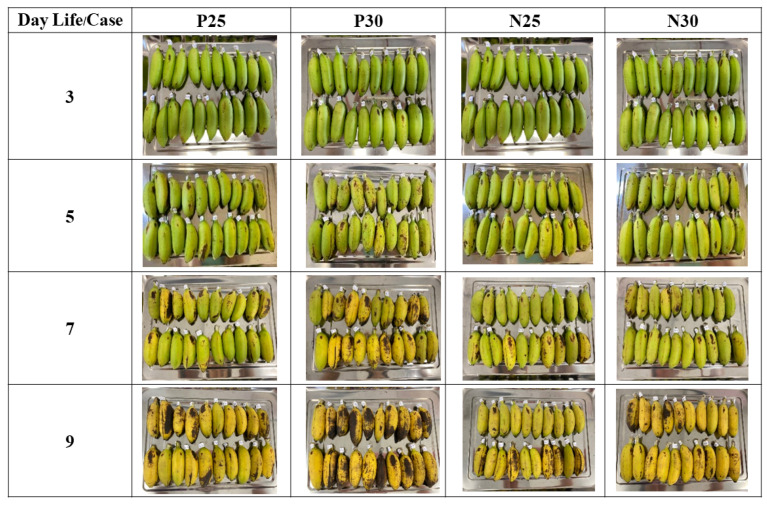
The four experimental groups used for data collection in this study are shown at Day 3, Day 5, Day 7, and Day 9 after harvesting. P25 = wrapped in wax paper and ripened at 25 °C, P30 = wrapped in wax paper and ripened at 30 °C, N25 = no wax paper and ripened at 25 °C, N30 = no wax paper and ripened at 30 °C.

This study has four experimental groups, which represent a matrix of two variables: the temperature during ripening, which was either 25 °C or 30 °C (based on local storage temperatures), and the presence or absence of wax paper wrapped around each individual banana. The four groups were called P25, P30, N25, and N30, where P means “paper used”, N means “no paper used”, and the numbers are the temperatures. Each experimental group consisted of 20 bananas (*n* = 20). The purpose of these experimental groups is not to find an optimal temperature or wrapping option, but rather to recognize that people handling bananas might store them at a variety of temperatures, either with or without some form of wrapping. Thus, the four experimental groups represent some normal variations that occur in actual everyday conditions. The four experimental groups and some snapshots of their ripening process are shown in Figure 3.

There were two cabinets where the bananas were ripened: one cabinet maintained at 25 °C for groups P25 and N25, and the other cabinet, which was maintained at 30 °C for groups P30 and N30. In the wax paper groups P25 and P30, each banana was individually wrapped. The purpose of wrapping these bananas was to investigate the effect of reducing circulation of the naturally produced ripening gas ethylene. Wax paper is a material readily available to people who handle bananas, and it does not promote decomposition of the bananas the way sealed plastic wrap can.

All 80 bananas (4 groups × 20 samples) were measured with a spectrometer and photographed with a smartphone once a day. They were measured for color by both methods daily until the banana skin color was substantially discolored. This produced a total of 9 days of data for all 80 bananas. The spectrometer measured all three CIE L*a*b* color values from the banana skin and the smartphone simply captured an image. RGB color data were later pulled from the phone images using MATLAB and then converted to CIE L*a*b* values. Thus, the nine-day data collection process yielded a total of 1440 records with L*a*b* values, consisting of 720 records from the spectrometer and 720 records from the smartphone.

### 2.2. Creating a Colorimetric Equation to Transform the Spectrometer Data

**Figure 4:**
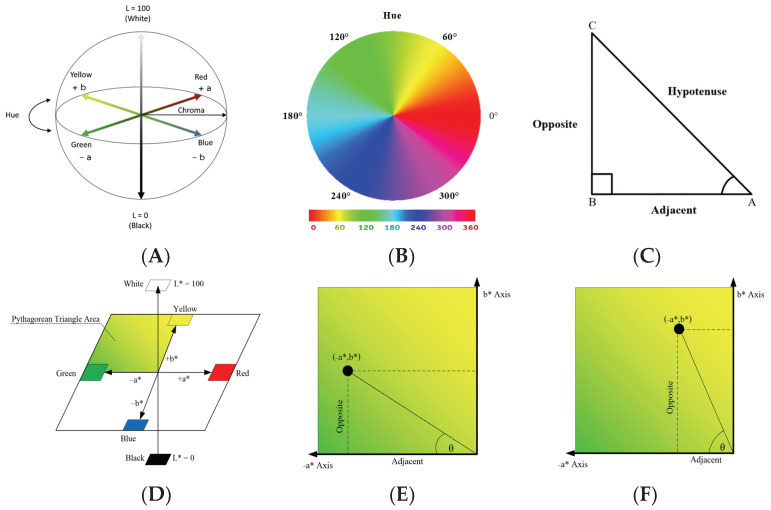
Elements related to the colorimetric equation in this study: (**A**) the CIE color space [16], (**B**) a color (hue) wheel [19], (**C**) a right triangle [22], (**D**) location of the hue plane between green and yellow, here called the Pythagorean Triangle Area (PTA), and (**E**,**F**) two examples of L*a*b* results plotted in the PTA.

The purpose of the colorimetric equation in this study is to convert CIE L*a*b* color data to a single variable representing hue, which will be θ. The CIE L*a*b* color system (CIELAB), shown in Figure 4A, is based on three axes with different colors at each end: (1) red and green, (2) yellow and blue, and (3) white and black. This system was developed in 1976 by the International Commission on Illumination (CIE), which is an organization devoted to international cooperation and exchange of information among its member countries on all matters relating to the science and art of lighting. In CIELAB, The L* axis indicates lightness, and the a* and b* axes indicate chromaticity. L* is represented as a vertical axis with values from 0 (black) to 100 (white). The a* axis represents red as +a* (positive) and green as −a* (negative). The yellow and blue components are represented on the b* axis as +b* (positive) and −b* (negative) values, respectively [16].

The θ values in this study will range from 0${}^{\circ }$ to 90${}^{\circ }$, and these represent angles between green and yellow/brown in the CIE L*a*b* color space. In a color wheel, shown in Figure 4B, the θ values correspond to the 90 degrees starting at hue angle = 150${}^{\circ }$ (dark green, θ = 0${}^{\circ }$) and finishing at hue angle = 40${}^{\circ }$ (brown, θ = 90${}^{\circ }$). Expressing and handling the hue as θ facilitates processing of collected data allows for creation of the eventual phone application.

The colorimetric equation in this study applies the CIE L*a*b* color system [16] together with the Pythagorean theorem [24]. Specifically, the Pythagorean theorem is applied to navigate the relationship between the −a* and +b* axes in CIE L*a*b*. The colorimetric equation here involves a right triangle. A right triangle consists of a hypotenuse (“h”) side, a base (“b”, or “adjacent”) side, and a perpendicular (“p”, or “opposite”) side, as shown in Figure 4C. “Adjacent” and “opposite” refer to the side’s relative position to the desired unknown angle. A right triangle is usually displayed with the base side horizontal at the bottom and the perpendicular side is displayed vertically. Trigonometric functions can be used to calculate the angles of a right triangle placed inside the hue plane of the CIE color space between green and yellow. This space will be referred to here as the Pythagorean Triangle Area (PTA), and it is shown in Figure 4D.

Spectrometer results from the 20 samples in each experimental group were averaged together. An example of a result is L* = 51.23, a* = −19.97, and b* = 56.35. The L* result is discarded here, because it is not related to hue. Next, the a* and b* values are plotted in the PTA, and their intersection is the location of that color. Figure 4E,F show two random samples after they have been plotted into the PTA.

Inside the Pythagorean Triangle Area, Figure 4E,F show how the plotted a* and b* coordinates define a right triangle with an unknown angle. This unknown angle will be the θ value used to represent banana skin hue in this study, and it will be called the angle of ripening. This angle of a right triangle could normally be calculated from the length of the opposite and adjacent sides by using the tan formula. However, because the current adjacent side is on the −a* axis, the result would be negative. Therefore, the equation for finding this angle is adjusted by using the absolute value of the −a* axis, as shown in Equation (1).

(1) tan(θ)=B/abs(A)

where θ is the angle of ripening, *A* is the value of a* (CIELAB), and *B* is the value of b* (CIELAB).

The CIELAB color values collected from the banana skins with the spectrometer are applied to Equation (1), which combines the CIELAB data with the Pythagorean theorem. Equation (1) will also be referred to as the colorimetric equation in this study. The result is θ (hue) values for all the spectrometer data, and this process will be referred to as Colorimetric Extraction from Color Data (CEFCD).

### 2.3. Creating a Colorimetric Model to Pull Data from the Smartphone Images

The colorimetric model in this study has two objectives: to capture only relevant color information from a smartphone image of a banana; and to adjust the brightness of the captured color information to a standardized level. The brightness needs to be standardized because the final phone application needs to be able to operate in uncontrolled lighting conditions, so the initial brightness of images will vary. The colorimetric model meets these two objectives using two processes: the object detection process and the energy calculation and illumination adjustment process. Each of these is explained below.

#### 2.3.1. Object Detection Process

The banana in each image is isolated from its background using the COCO-SSD object detection model [17]. COCO-SSD is able to detect and classify 90 different classes of objects in an image based on its previous training with an image database called the COCO Dataset [25]. That dataset includes bananas, so COCO-SSD has the ability to detect a banana in an image. The performance of the COCO-SSD model in the context of this study will be evaluated in two ways. First, the ability of COCO-SSD to correctly demarcate the object will be evaluated using the Intersection Over Union (IOU) method [26] to calculate the average and standard deviation (SD) of the overlapping area between a rectangular demarcation of the banana’s dimensions generated by COCO-SSD and a similar rectangular demarcation generated by the researcher using visual examination together with the LabelImg [27] graphical image annotation tool. Second, the accuracy rate of COCO-SSD in correctly identifying the object as a banana will be calculated.

#### 2.3.2. Energy Calculation and Illumination Adjustment Process

The ultimate purpose of this procedure is to adjust images taken by the application user under varying light conditions so that all images will adopt a uniform brightness that is based on the illumination environment of the original laboratory lightbox pictures. This is carried out through the following four steps.

**Figure 5:**
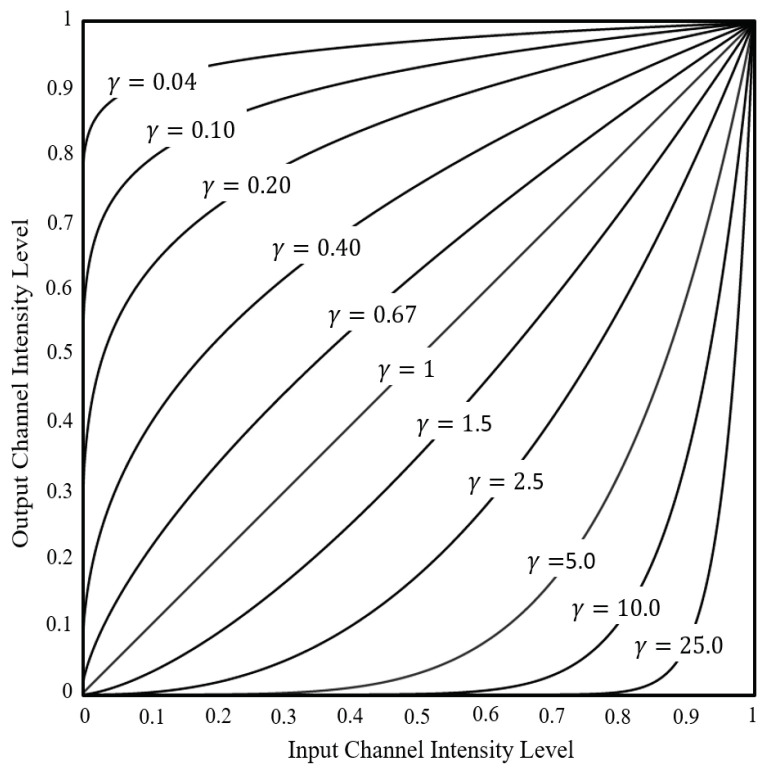
An example of how altering γ can affect channel intensity level (when c is fixed at 1), as modified from [29].

Step 1: First, the overall brightness (OB) of each standard lightbox image (LI) is measured. The formula used here for calculating the OB of any image is based on the Minkowski norm formula [28], and it is shown in Equation (2).
  (2) $$OB_{\left(any\_image\right)}\;=\;\left(R/3\right){\left[\frac{\sum_{x=1}^{M}\sum_{y=1}^{N}{\left(R\left(x,y\right)\right)}^{p}}{MN}\right]}^{1/p}+\left(G/3\right){\left[\frac{\sum_{x=1}^{M}\sum_{y=1}^{N}{\left(G\left(x,y\right)\right)}^{p}}{MN}\right]}^{1/p}+\left(B/3\right){\left[\frac{\sum_{x=1}^{M}\sum_{y=1}^{N}{\left(B\left(x,y\right)\right)}^{p}}{MN}\right]}^{1/p}$$
  where $OB_{\left(any\_image\right)}$ is the overall brightness of all the pixels in the image; *M* and *N* are the vertical and horizontal pixel counts of the image, respectively; *R*, *G*, and *B* are the intensity values of the red green and blue channels, respectively, in one pixel of the RGB image; and *p* is the weight of each gray value in the brightness value (*p* = 2).
Step 2: Now, the OB values of all the LIs will be averaged. The average OB of all the LIs is calculated using Equation (3).
  (3) $${\bar{OB}}_{\left(LI\right)}\;=\;\frac{\sum_{i=1}^{N}OB_{\left(LI(any\_image)\right)}}{N}$$
  where ${\bar{OB}}_{\left(LI\right)}$ is the average overall brightness of all the lightbox images (LI), and *N* is the total number of lightbox images.
  The Standard Brightness (SB) to which each incoming Unadjusted Image (UI) from the app will be adjusted is a collection of data consisting of 10 components, called Brightness Components (BC) here; the ${\bar{OB}}_{\left(LI\right)}$, or average overall brightness of the LIs, is only one of these BCs. The average brightness, contrast, and gradient of each color channel from all pixels are also calculated here. That means that there are an additional nine BCs: Brightness of channel R (BR), Brightness of channel G (BG), Brightness of channel B (BB), Contrast of channel R (CR), Contrast of channel G (CG), Contrast of channel B (CB), Gradient of channel R (GR), Gradient of channel G (GG), and Gradient of channel B (GB) [28]. Although here in Step 2, the 10 BCs have been used to represent the brightness of all LIs as SB, the same 10 BCs can be calculated for any image to similarly represent its brightness, as will be seen in the following Step 3.
Step 3: Once the ${\bar{OB}}_{\left(LI\right)}$ and other BCs of the LIs have been calculated to establish the SB, the overall brightness of an unadjusted image ($OB_{\left(UI\right)}$) will now be calculated, using Equation (2) as before. The other 9 BCs will also be calculated from the UI here in Step 3.
Step 4: After the $OB_{\left(UI\right)}$ is known, the UI will go through the Power-Law method, which, in this study, will create 100 alternative image versions, called Candidate Images (CI), each with a different brightness adjustment, based on all possible combinations of 10 different C constants (set at 1–10) and 10 different gamma (γ) constants (which vary, depending on the UI, as will be explained later). This method is shown in Equation (4). The Power-Law method adjusts brightness by changing the R, G, and B channel intensity levels in each pixel, depending on the interaction of C and gamma. Figure 5 shows an example of how altering gamma can affect channel intensity level.
  (4) $$CI\;=\;c*UI^{\gamma }$$
  where CI is the Candidate Image (CI), UI is the Unadjusted Image (UI), *c* is a constant (positive number), and γ is the gamma constant (positive number).

The Power-Law method [30] can increase or decrease the brightness of the UI to varying degrees by adjusting c and gamma, but the challenge when using this method is to find the optimal c and gamma. A process of trial and error is used here. More specifically, gamma alone will determine whether brightness increases or decreases, but c can affect the amount of increase or decrease. This study uses the results of the OB calculations to find out if brightness needs to be increased or decreased, and the answer will decide which of two sets of gammas is used for optimization, thus halving the time required to determine the best gamma. Whether the brightness needs to be increased or decreased is found as follows:

If $OB_{\left(UI\right)}$ > ${\bar{OB}}_{\left(LI\right)}$, that means the UI has **higher** overall brightness than the LIs. In this case, a set of gammas greater than zero will be used for optimization, as follows: *c* = [1, 2, 3, 4, 5, 6, 7, 8, 9, 10] and γ = [1, 2, 3, 4, 5, 6, 7, 8, 9, 10])
If $OB_{\left(UI\right)}$ < ${\bar{OB}}_{\left(LI\right)}$, that means the UI has **lower** overall brightness than the LIs. In this case, a set of gammas less than or equal to zero will be used for optimization, as follows: *c* = [1, 2, 3, 4, 5, 6, 7, 8, 9, 10] and γ = [0, 0.1, 0.2, 0.3, 0.4, 0.5, 0.6, 0.7, 0.8, 0.9])

In either case above, every possible combination of the specified c and γ values was applied to one UI using the Power-Law method, resulting in 100 alternative versions (10 possible c values × 10 possible γ values) from each UI. These alternative versions are the Candidate Images (CIs) mentioned earlier. They have this name because from these 100 candidates, the optimal c/γ combination will be selected. In order to make that selection, all the BCs of each CI are compared with all the average BCs of the LIs, i.e., the Standard Brightness, and the differences are summed together using the city block distance method [31] to establish the Brightness Difference (BD) between each CI and the Standard Brightness. An ideal BD would be 0, indicating that the brightness of that CI matches the Standard Brightness. The Brightness Difference of each candidate image was calculated using Equation (5).

(5) $$BD_{\left(any_{\_}c,\gamma \right)}\;=\;|{\bar{OB}}_{\left(LI\right)}-OB_{{\left(CI\right)}_{\left(c,\gamma \right)}}|+|BRofSB-BRofCI_{\left(c,\gamma \right)}|+|CRofSB-CRofCI_{\left(c,\gamma \right)}|+...+|GBofSB-GBofCI_{\left(c,\gamma \right)}|$$

where $BD_{\left(any_{\_}c,\gamma \right)}$ is the Brightness Difference between the Candidate Image that resulted from *c* and γ, and the Standard Brightness; ${\bar{OB}}_{\left(LI\right)}$ is the average overall brightness of all the LIs; $OB_{{\left(CI\right)}_{\left(c,\gamma \right)}}$ is the overall brightness of the Candidate Image that resulted from *c* and γ; BRofSB is the brightness of the red channel of the Standard Brightness; $BRofCI_{\left(c,\gamma \right)}$ is the brightness of the red channel of the Candidate Image that resulted from *c* and γ; CRofSB is the contrast of the red channel of the Standard Brightness; $CRofCI_{\left(c,\gamma \right)}$ is the contrast of the red channel of the Candidate Image that resulted from *c* and γ; GBofSB is the average gradient of the blue channel of the Standard Brightness; and $GBofCI_{\left(c,\gamma \right)}$ is the average gradient of the blue channel of the Candidate Image that resulted from *c* and γ.

After the Brightness Difference has been calculated for all the Candidate Images of the Unadjusted Image, the CI with the lowest BD will be selected as optimal and thereafter be called the Adjusted Image for further use, because the brightness of the UI has been adjusted as close as possible to the Standard Brightness of the lightbox images. This methodology utilizing the Power-Law method in combination with the energy calculation process based on the Mosashi norm formula is referred to here as Automatic Power-Law Transformation (APLT).

The effectiveness of APLT will be validated by creating 2 new copies of each of the original 720 unadjusted smartphone images and intentionally corrupting their brightness either upwards or downwards to confirm that APLT is later able to accurately correct their brightness. The images thus used in this experiment will be called Validation Images (VIs). Half of the VIs (720 images) will have their brightness decreased by 50% using the MATLAB “Imadjust” function (RGB,[],[],0.5) [32]. The second half of the VIs (the other 720 images) will have their brightness increased by 50%, also using the Imadjust function (RGB,[],[],1.5). All 1440 VIs will then go through APLT. Finally, the resulting brightness of the VIs will be compared to the Standard Brightness.

#### 2.3.3. Removing the Image Background

An original image from the smartphone has now passed through object detection to isolate the banana from the background as well as the energy calculation and illumination adjustment process to convert the image to Standard Brightness. However, there are still some remnants of the background, because the banana was isolated as a rectangle. The current step will remove remnants of the background by first finding the optimal global threshold of the AI using Otsu’s method [33] and then setting each background pixel to black and each non-background pixel to white by comparing the threshold value to the intensity of the pixel in a grayscale version of the image, as shown in Equation (6).

(6) $$g\left(x,y\right)=\left\{\begin{array}{cc} 1, & \mathrm{if}\;\mathrm{intensity}\;\mathrm{of}\;f(x,y)>\;T\\ 0, & \mathrm{if}\;\mathrm{intensity}\;\mathrm{of}\;f(x,y)\leq \;T \end{array}\right.$$

where g(x,y) is the processed pixel at (x,y) that has been set to black or white; f(x,y) is the unprocessed pixel at (x,y) in the grayscale image; and *T* is a threshold determined separately by Otsu’s Method.

#### 2.3.4. Convert Adjusted RGB Data to L*a*b* Data

Remember that the spectrometer reported on three points along the length of the banana, and data from those points were averaged to resemble L*a*b* readings from a single point for use in the colorimetric equation. However, the current adjusted image is still an image with many pixels. Thus, these many pixels similarly need to be averaged together to resemble one pixel, after which that averaged RGB information will be converted to L*a*b* for use in the colorimetric equation. To do this, all the red channel values of non-black pixels in the current image will be averaged together to obtain one average red channel value. In the same way, the green and blue channel values will each be averaged from all non-black pixels. The set of these three channel averages are like RGB values from a single point.

The last step here is to convert the averaged RGB values to CIE L*a*b* color values and then send the L*a*b* color values to the colorimetric Equation (1) to obtain the resulting θ value. The complete process of converting the raw smartphone image to a θ value is referred to here as colorimetric extraction from image (CEFI).

### 2.4. Colorimetric Model Adjustment

Data obtained from the spectrometer are considered better than data obtained from a smartphone, because the smartphone image is subject to uncontrolled lighting, whereas the spectrometer is not. In order to look for any consistent variation between θ values derived from colorimetric extraction from color data (CEFCD) and θ values derived from the colorimetric extraction From image (CEFI), results from the two methods were compared. If there is a consistent error rate, an error constant can be incorporated into the colorimetric model as an adjustment to bring the smartphone result closer to the spectrometer result. To test for this error rate, the original lightbox images were processed through CEFI, as if they were smartphone images produced without a lightbox. The θ values from those results were then compared, banana by banana, to the θ values from the original spectroscope data (using the same bananas) that had passed through CEFCD. This comparison was done for all the experimental groups (P25, P30, N25, and N30) and on all the experiment days. The error rate was calculated using Equation (7).

(7) $$E_{\theta }\;=\;\frac{\sum_{i=1}^{N}|\theta_{1,P25}CEFCD-\theta_{1,P25}CEFI|+|\theta_{2,P25}CEFCD-\theta_{2,P25}CEFI|+...+|\theta_{n,N30}CEFCD-\theta_{2,N30}CEFI|}{M/2}$$

where $E_{\theta }$ is the error rate between the θ values of colorimetric extraction from color data (CEFCD) and colorimetric extraction from image (CEFI) for all experimental groups and all experiment days; $\theta_{1,P25}CEFCD$ is the θ value of colorimetric extraction from color data (CEFCD) on Day 1 for P25; $\theta_{1,P25}CEFI$ is the θ value of colorimetric extraction from image (CEFI) on Day 1 for P25; and *M* is the total number of the θ values.

If the error rate is found to be significant and consistent, then it can be added as an error constant in the original colorimetric equation. This modified calculation is shown in Equation (8).

(8) $$tan\left(\theta \right)\;=\;B/abs\left(A\right)+E_{\theta }$$

where θ is the angle of ripening, *A* is the value of a* (CIELAB), and *B* is the value of b* (CIELAB).

The angle of ripening from the final version of the colorimetric equation will be compared with the daily visual observations of the banana skins to map out equivalents between theta values and the remaining days For consumption (RDFC).

### 2.5. Application Development

The Automatic Banana Ripeness Indicator (ABRI, pronounced like “Aubrey”) application was developed and designed following the principles of System Development Life Cycle (SDLC) [34] for software development. The application has a responsive design that automatically adjusts for different size screens and viewports [35], and it works on any mobile device running any operating system. The application has two main screens, behind which all the image processing procedures described to this point run. The first screen is for photographing a banana, displaying the photo, and initiating colorimetric analysis. The second screen displays the results of the analysis, indicating the ripeness stage of the banana and an estimate of the remaining number of days in which it can be consumed. The application will be evaluated for usability and validity by knowledgeable people in relevant fields.

## 3. Results

In the course of this research to develop a colorimetric equation and colorimetric application to conveniently identify the ripening stages of Kamphaeng Phet lady finger bananas, an important challenge to overcome is the variety of light environments in which the app users will ultimately photograph bananas. This section presents the results of the three experiments described earlier to test and validate three important steps of the development process. Information on the finished app is also provided here.

### 3.1. The Object Detection Process Experiment Result

**Figure 6:**
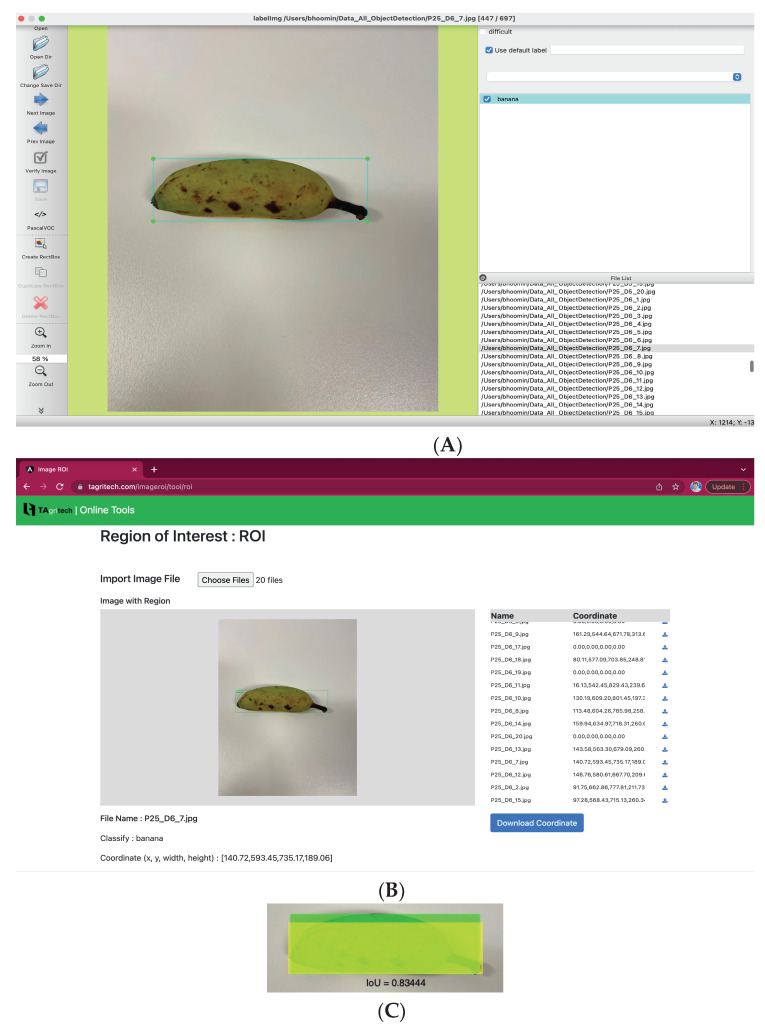
Sample detection results showing (**A**) demarcation using the graphical image annotation tool LabelImg, (**B**) demarcation using the COCO-SSD model, and (**C**) the two demarcated areas superimposed to show the amount of overlap.

The banana in each of the 720 banana smartphone photos from the data collection process was demarcated with a rectangular in 2 ways: first, it was performed manually by using the LabelImg graphical image annotation tool. Then, it was automatically determined using the COCO-SSD object detection model. The results of the two methods were compared. A sample output from LabelImg is shown in Figure 6A, and a sample output from COCO-SSD is shown in Figure 6B.

**Figure 7:**
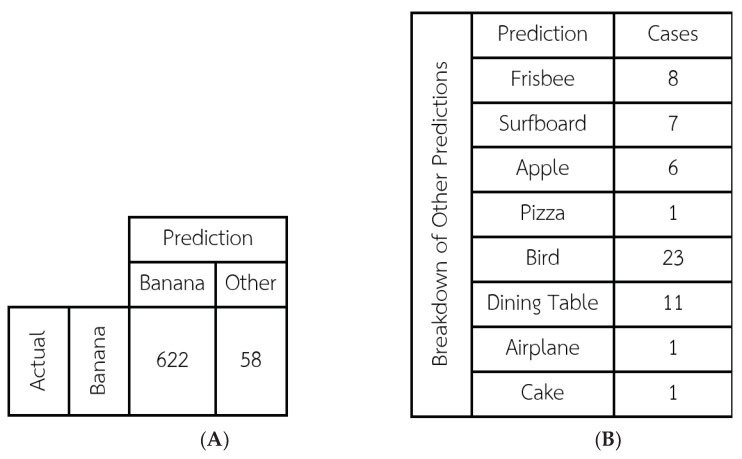
The classification results of the COCO-SSD model when applied to the 720 banana images in this study: (**A**) the number of correct and incorrect predictions by COCO-SSD and (**B**) a breakdown of the predictions other than “Banana”.

In order to evaluate the model’s ability to demarcate bananas accurately, the results of the two demarcation procedures described above were compared using the Intersection Over Union (IOU) method, which calculates the area where the two rectangles intersect divided by the total area of the united rectangles (counting the intersection area only once). The average IOU result for all 720 images was 0.857 with a S.D. of 0.137. These two figures show that the model can demarcate bananas at high levels of effectiveness and reliability. Next, the rate at which the COCO model was able to correctly classify (identify) the photo as a banana was calculated. The classification results are shown in the Figure 7.

The COCO-SSD model correctly classified 622 of the 720 images as a banana, corresponding to a high accuracy rate of 91.94%. Visual examination of the photos that were misclassified suggests that the issues were related variously to: the distance at which the photo was taken (in the cases of Frisbee, Surfboard, and Apple), a light reflection in the photo (Dining Table), and a significant amount of dark spots on the banana (Pizza, Bird, Airplane, Cake). The distance issue is addressed in the application, which will constantly display the current classification prediction. The user can move closer or further away until COCO-SSD displays the word “Banana”, and then take the photo.

The results of the demarcation and classification experiments indicate that the COCO-SSD algorithm functions well in this study for the intended purposes.

### 3.2. Energy Calculation and Illumination Adjustment Process Experiment Result

Table 1 shows all 10 of the Brightness Components (BCs) of the Standard Brightness (SB), which was calculated and averaged from all the lightbox images. The goal of this evaluation was to calculate the Brightness Components of the intentionally brightened or darkened Validation Images (VIs) after they passed through the energy calculation and illumination adjustment process and then compare those BCs of the adjusted VIs to the BCs of the SB, since the goal of the Automatic Power-Law Transformation (APLT) for illumination adjustment is to move the BCs of the VIs as close as possible to the BCs of the SB.

**Figure 8:**
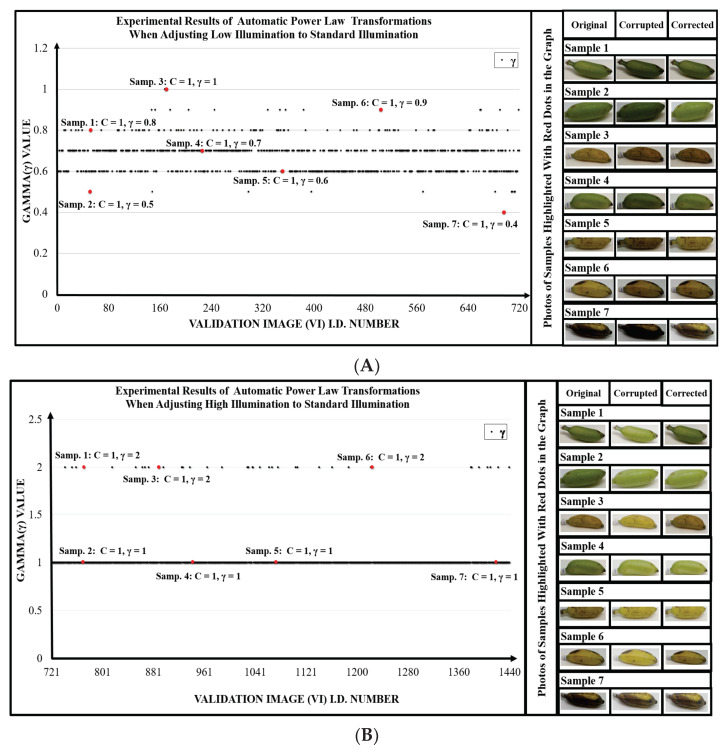
Optimized gamma values for the Validation Images after Automatic Power-Law Transformation (APLT), showing results for (**A**) images that were intentionally darkened and (**B**) images that were intentionally brightened. The optimized C value was always 1.

Remember that various gamma and c values were trialed to adjust the BCs of each VI, and the particular combination of gamma and c that gave the best result for each image was selected by Equation (5). Figure 8 shows a graph of all the finally selected gamma and c values that were required to adjust the Brightness Components of the Validation Images as close as possible to the Brightness Components of the Standard Brightness.

As one would expect, the intentionally darkened VIs had an overall brightness (OB) value less than the OB of the established Standard Brightness (SB), and the optimized gamma used to brighten these images to the SB varied between 0.4 and 1, depending on the image, as shown in Figure 8A. In the case of the VIs that were intentionally brightened, their OB was greater than the OB of the SB, so those VIs were adjusted to the SB using optimized gamma values between 2 and 3, as shown in Figure 8B.

In order to compare the brightness components of the adjusted validation images to the Standard Brightness components and thereby evaluate the accuracy of the illumination adjustment process, the overall brightness (OB) component was disregarded, because it is a composite value. The remaining 9 brightness components each have possible values ranging from 0 to 255. Therefore, if each of a single adjusted validation image’s 9 BCs are subtracted from the corresponding SB BC, the maximum difference would be 255. A difference of 255 would indicate a completely wrong adjustment, and a difference of 0 would indicate a perfect adjustment. Therefore, the difference of the components divided by 255 and then multiplied by 100 will give the accuracy of that particular image’s adjustment.

This calculation was carried out for all 1440 adjusted validation images, and the average accuracy and SD were then found. The result is that the validation images were adjusted to Standard Brightness with an accuracy of 95.72% (S.D. 5.11). Thus, the illumination adjustment process developed in this research was very effective at adjusting and standardizing the brightness of the test images that were intentionally lightened and darkened for this evaluation, and the process is a good tool for adjusting and standardizing the brightness of images taken by users of the app under uncontrolled light conditions.

### 3.3. The Colorimetric Equation Creation Experiment Results

**Figure 9:**
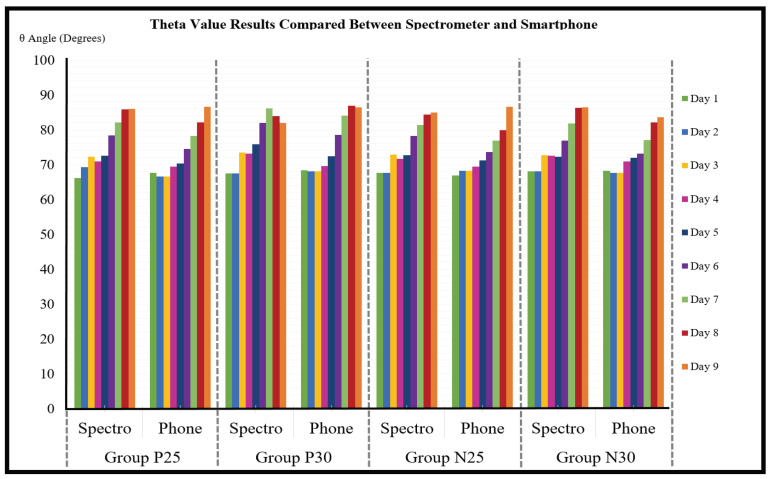
Theta results from the spectrometer are compared with those from the corresponding smartphone images in all four experimental groups and on nine all experiment days.

The theta values calculated from the spectrometer data were compared to the theta values calculated from the corresponding smartphone lightbox images for all four experimental groups and for all nine experiment days, and the results are shown in Figure 9.

**Figure 10:**
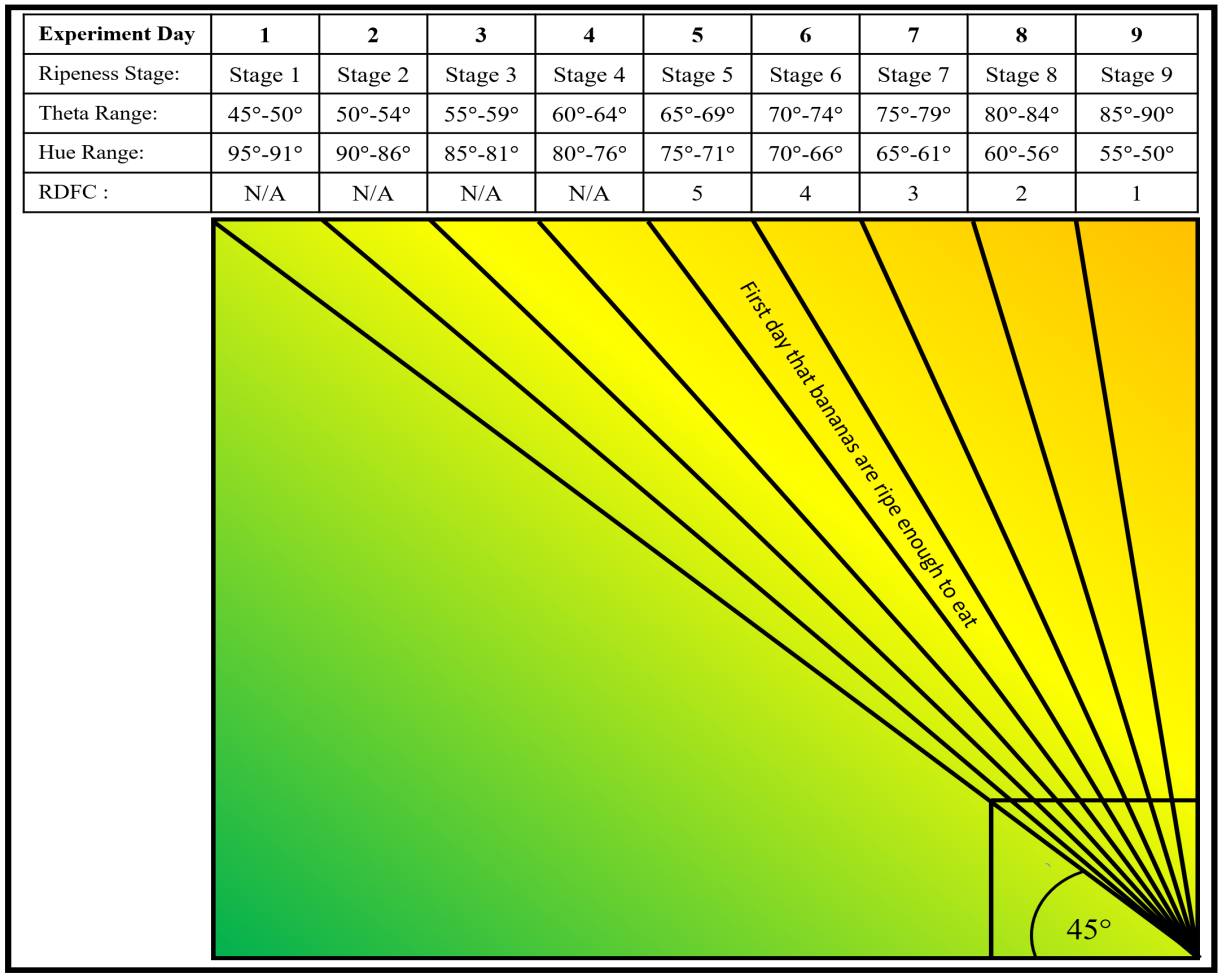
The relationships between experiment day, ripeness stage, theta range, hue range, and remaining days for consumption (RDFC) for all experimental groups averaged together.

The average error rate of the smartphone theta values compared to the spectrometer theta values was calculated across all four experiment groups and all nine experiment days using Equation (7), and the result was 2.8° ± 0.2°. Therefore, an error constant of 2.8 was incorporated into Equation (8). Figure 10 shows the relationships between experiment day, ripeness stage, theta range, hue range, and remaining days For consumption (RDFC) for all experimental groups averaged together. As in other related studies on banana ripening, the passing of 1 Ripeness Stage takes approximately 24 h. The observed theta ranges within each Ripeness Stage varied from 4 to 5°, and the observed hue range within each Ripeness Stage also varied from 4 to 5°. The first day that the bananas were ripe enough to eat was experiment Day 5, corresponding to Ripeness Stage 5, and they remained edible for a total of five days (Ripeness Stage 5–9) before they became overripe. The remaining days for consumption (RDFC) status includes the current day. Bananas cannot be cut before Stage 1, because they will only shrivel, not ripen, and bananas cannot be eaten after Stage 9, because they will be overripe.

### 3.4. Application Development

**Figure 11:**
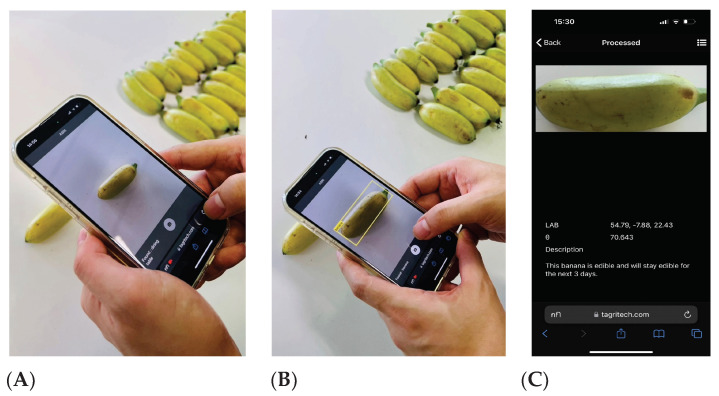
An example of the ABRI app in use: (**A**) The banana is brought into the camera frame, (**B**) the banana is detected by the app, and (**C**) analysis results on the ripeness of the banana are displayed.

The research in this study was developed into an easy-to-use progressive web application that conveniently processes banana photos directly from the user’s mobile device camera. The application is accessible from any mobile device and any operating system, and it is called the Automatic Banana Ripeness Indicator (ABRI, pronounced like “Aubrey”). Figure 11 shows an example of the ABRI app in use.

When the ABRI icon is clicked, the application immediately opens the device camera to accept a banana photo. When a banana is brought into the camera frame, the app will automatically detected it and highlighted it with a yellow detection box. When the user then takes the photo, it is automatically processed and analyzed as described earlier in this study, and the user is taken immediately to the results display page.

After completion, the application was evaluated for usability and accuracy by five knowledgeable people: two people in the banana business and three biologists. The five evaluators gave ABRI good marks, with an average 4.51 points out of 5 possible points across all the evaluation questions. The ABRI application is now ready to use to conveniently and accurately identify the ripening stage of Kluai Khai bananas.

## 4. Discussion

The results of the colorimetric equation and colorimetric detection model for identification of the ripening stage of ‘Kluai Khai’ Banana were well aligned with the spectrometer results, providing an effective level of accuracy. A central strength of the application lies in its ability to accept photos taken in a variety of lighting environments, because the Automatic Power-Law Transformation (APLT) method automatically adjusts the image brightness to an established standard. This is a valuable feature, because unstable lighting is a challenge that always affects the field of image processing. An overview of the Automatic Banana Ripeness Indicator (ABRI) developed in this current study in comparison with previous studies is shown in Table 2, looking at key features, platforms, and accuracy.

Each of the studies compared in Table 2 was based on a particular platform and had its own objectives; thus, the key features vary. A study can be “contact” or “non-contact”. A contact study involves a sensor actually contacting the skin of the banana to collect color information [19], and this can potentially bruise the banana. Non-contact studies use a camera to collect color information without contacting the skin of the banana [7,9,10,11,12,13,14,15]. Studies can also be divided by platform, with some studies running on a computer [7,9,11,12,13] and other studies running on a smartphone [10,14,15]. A computer can provide higher computing power, while a smartphone can provide more convenience and portability. That said, software with special functionality such as object detection and illumination adjustment can be added to smartphones to compensate for some shortcomings compared to a computer. Unfortunately, both computer and smartphone platforms that are used in an uncontrolled environment are subject to unstable lighting. This study overcame that challenge with the APLT method. The research of Zhu, L. et al. [10] included object detection using the Yolo algorithm, and it classified bananas into four ripening stages using the SVM algorithm. However Zhu et al. were forced to use only lightbox images because no way to adjust for uncontrolled lighting was included. Intaravanne, Y. et al. [14] applied frequency domain analysis to classify bananas into three ripening stage but they did not include lighting adjustment either. The study of Kipli, K. et al. [15] was similar to that of Intaravanne, Y. et al., but it applied data mining to accomplish classification of ripening stages. The main characteristics of the current study that set it apart from others are the use of the APLT method and the colorimetric equation as well as the remaining days for consumption feature that is outputted by the ABRI app. The fact that ABRI is written as a progressive web application also adds flexibility and makes installation unnecessary.

## 5. Conclusions

The Automatic Banana Ripeness Indicator ABRI smartphone application developed here is an accurate and robust tool that quickly, conveniently, and reliably provides the user with any LFB’s ripening stage and the remaining days for consumption. At present, it has the limitation of processing this one variety of banana that is important in this area. Going forward, the authors plan to expand upon these analytical capabilities to enable processing of multiple varieties of bananas. The techniques described here also lend themselves to adaptation for developing related analysis of other important agricultural products in the future.

## Figures and Tables

**Table 1:** All 10 Brightness Components of the Standard Brightness, which was calculated and averaged from all the lightbox images.

OB	Brightness			Contrast			Average Gradients		
	r	g	b	r	g	b	r	g	b
6.14	117.91	117.44	67.88	45.44	45.70	71.96	18.02	18.54	19.35

**Table 2:** Overview of the Automatic Banana Ripeness Indicator (ABRI) in comparison with previous studies, looking at key features, platforms, and accuracy.

Study	Contact	CE	Platform	RS	RDFC	Images	Classifier	OA(%)
Current Study (ABRI)	No	No	Mobile	9	Yes	Yes	Yolo + APLT + CIEPT	92.88
[7]	No	Yes	Computer	No	Yes	Yes	CIE-L*a*b*	No
[8]	Yes	Yes	Computer	7	No	No	DA- meter	No
[9]	No	Yes	Computer	3	No	Yes	Size + MCI	85.00
[10]	No	Yes	Mobile	4	No	Yes	Yolo + SVM	96.40
[11]	No	Yes	Computer	4	No	Yes	Yolo + CNN	71.95
[12]	No	Yes	Computer	3	No	Yes	GLCM + NN	75.67
[13]	No	Yes	Computer	14	No	Yes	CNN	91.00
[14]	No	Yes	Mobile	3	No	Yes	SA	No
[15]	No	Yes	Mobile	3	No	Yes	DL	No

Contact = There is physical contact with the banana skin, CE = Controlled Environment, RS = Ripening Stage, RDFC = Remaining Days For Consumption, OA = Overall Accuracy, APLT = Automatic Power-Law Transformation, CIEPT = CIE L*a*b* Color Scale with Pythagorean Theorem, MCI = Mean Color Intensity, SVM = Support Vector Machines, CNN = Convolutional Neural Networks, GLCM = Gray Level Concurrence Matrix, SA = Spectral Analysis, and DM = Data Mining.

## Data Availability

Raw data can be requested through the corresponding author.
